# Effect of Liuweibuqi capsules on the balance between MMP-9 and TIMP1 and viability of alveolar macrophages in COPD

**DOI:** 10.1042/BSR20170880

**Published:** 2017-09-19

**Authors:** Chengyang Wang, Huanzhang Ding, Xiao Tang, Zegeng Li, Lei Gan

**Affiliations:** 1 Department of Traditional Chinese Medicine, The First Affiliated Hospital, Anhui Medical University, Hefei 230022, China; 2 Graduate School, Anhui University of Chinese Medicine, Hefei 230022, China; 3 Anhui Academy of Chinese Medicine, Hefei 230022, China

**Keywords:** chronic obstructive pulmonary disease, inflammatory cytokines, Liuweibuqi capsules, MMP-9, TIMP1

## Abstract

The present study aims to investigate the effect of Liuweibuqi (LWBQ) capsules on the expression of matrix metalloproteinase (MMP)-9 and TIMP1 and cell viability of alveolar macrophages (AMs) in chronic obstructive pulmonary disease (COPD). Rats were randomly divided into normal control (NC) group, model control (MC) group, Jinshuibao (JSB) group, spleen aminopeptidase (PAT) group, and low dose of LWBQ (LWBQ low), mid dose of LWBQ (LWBQ mid), and high dose of LWBQ (LWBQ high) group (*n*=10). Lung function was measured with a spirometer. Serum cytokines including tumor necrosis factor-α (TNF-α) and interleukin-6 (IL-6) were detected using ELISA. The expressions of MMP-9 and TIMP1 were detected by quantitative real-time PCR (qRT-PCR) and Western blot. MTT assay and flow cytometry were used to measure cell viability and apoptosis. Compared with the NC group, body weight and lung function were reduced in the MC group. In addition, the serum levels of IL-6 and TNF-α were higher in the MC group than those in the NC group. The expression of MMP-9 protein in the AMs from rats was higher, and TIMP1 protein was lower in the MC group compared with the NC group. After LWBQ capsules treatment, compared with the MC group, the expression of inflammatory cytokines and MMP-9 were lower and TIMP1 was higher. Moreover, after LWBQ-medicated serum treatment, the release of inflammatory cytokines was reduced from AMs. Besides, LWBQ-medicated serum decreased the expression of MMP-9 and increased the expression of TIMP1 and cell viability compared with those in MC group. In conclusion, LWBQ capsules can inhibit the release of inflammatory cytokines, promote cell viability in AMs, and regulate the expression of MMP-9 and TIMP1.

## Introduction

Chronic obstructive pulmonary disease (COPD) is one of the leading causes of deaths worldwide [1]. Additionally, COPD is characterized by persistent airflow obstruction, which results from inflammation and remodeling of the airways [2]. The most critical risk factor for the development of COPD is cigarette smoking [3]. Treatment of COPD includes medications and non-pharmacologic interventions [4]. However, many COPD patients remain symptomatic regardless of medical therapy [5]. Therefore, strategies that may provide quality healthcare for COPD patients are required.

Liuweibuqi (LWBQ) capsules are a traditional Chinese medicine (TCM). It includes Renshen (*Radix Ginseng*), Huangqi (*Radix Astragali Mongolici*), Yuzhu (*Rhizoma Polygonati odorati*), Yizhi (*Fructus Alpiniae Oxyphyllae*), Chenpi (*Pericarpium Citri Reticulatae*), and Rougui (*Cortex Cinnamomi Cassiae*) [6]. Our previous studies have demonstrated that LWBQ capsules can reduce the release of inflammatory cytokines [6].

Alveolar macrophages (AMs) are resident lung macrophages, and present the first line of encountering inhaled substances [7]. Notably, AMs repress excessive inflammation through the strong inhibition of local immune cells, such as T lymphocytes [8]. Smoking leads to AM defects in response to pathogen [9]. In COPD, the persistence of inflammation and exhausted immune cells impair the phagocytic capacity of AMs [10]. The AM is a very interesting target for our investigations since it has been shown that inhibition of matrix metalloproteinase (MMP)-9 activation leads to abolished ability of macrophages to migrate to the site of inflammation [11].

MMP-9, one of the members of the MMP family, is a gelatinase that has been implicated in the pathogenesis of sepsis [12], COPD [13], atherosclerosis [14], and tumor formation and metastasis [15]. Additionally, increasing studies have found elevated serum levels of MMP-9 with many chronic inflammatory conditions including COPD [13,16]. Besides, MMP-9 is strictly regulated by its specific inhibitor of metalloproteinase 1 (TIMP1), which controls its proteolytic activity [17]. Imbalance between MMPs and TIMPs may cause excessive degradation of tissue, a condition that is often related to chronic inflammatory diseases [18]. Thus, the balance between MMP-9 and TIMP1 has emerged as a novel disease marker as well as a therapeutic target.

The present study aims to investigate the effect of LWBQ capsules on the expression of MMP-9 and TIMP1 and cell viability of AMs in COPD.

## Materials and methods

### Experimental animals

Male Sprague–Dawley (SD) rats (180–200 g) were purchased from the Experimental Animal Center of Anhui Province (Hefei, China). All animals were housed in specific pathogen-free (SPF) conditions, and given access to water and food *ad libitum*. The study protocol was approved by the Ethics Committee of The First Affiliated Hospital, Anhui Medical University. All animal experimental procedures were approved according to the guidelines of the Care and Use of Laboratory Animals by the National Institute of Health, China.

### Model establishment and grouping

Rats were randomly divided into seven groups, with ten rats in each group: normal control (NC) group, model control (MC) group, Jinshuibao (JSB) group, spleen aminopeptidase (PAT) group, low dose of LWBQ (LWBQ low) group, mid dose of LWBQ (LWBQ mid) group, and high dose of LWBQ (LWBQ high) group. Apart from the NC group, all rats were anesthetized with 10% chloral hydrate and their tracheas exposed to 200 μl of 1 mg/ml lipopolysaccharide (LPS, Sigma, U.S.A.). Subsequently, the rats were placed in a chamber with smoke from ignition of 50 g sawdust and 0.682 g cigarette tobacco mixture (Chuzhou, China; tar 13.5 mg/g and nicotine 0.48 mg/g). The rats were exposed to the smoke for 30 min per day for 28 days to establish the rat models [6].

### Drug treatment

Drug treatments started from day 28. The NC and MC groups were given 0.9% physiological saline (10 ml/kg) by gastric perfusion. The JSB and PAT groups were respectively administrated via gastric perfusion of JSB capsules (0.495 g/kg) and PAT (0.33 mg/kg) for 30 days. The LWBQ groups were administrated via gastric perfusion of LWBQ low capsules (0.2 g/kg), LWBQ mid (0.4 g/kg), and LWBQ high (0.8 g/kg) for 30 days. Then, rat body weight and respiratory rate were monitored. In addition, we sampled 5 ml of their abdominal aortic blood. The blood was centrifuged at 1000×***g*** for 5 min and serum was collected and stored at –80°C for later cytokine analysis.

### Evaluation of pulmonary function

Pulmonary function was observed by forced vital capacity (FVC) and average expiratory flow, which was calculated by dividing FVC by the value for forced expiratory flow in 0.3 s (FEV_0.3_) and multiplying by 100%. In addition, expiratory resistance (Re) was assessed. These measurements were obtained using the pulmonary function test apparatus for small animals 30 days after drug treatment.

### AM isolation

After drug treatment, the rats were killed by deep anesthesia with isoflurane. Bronchoalveolar lavage (BAL) fluid was collected [19] by instilling 1 mM EDTA/PBS into the lungs through a tracheal cannula using 0.5 ml solution five times, for a total of 2.5 ml. Following collection of the BAL fluid, AMs were isolated by centrifugation at 2400 rpm (1000 ***g***) for 10 min at 4°C [20]. Cells were resuspended in 1 ml RPMI-1640 mediaum(Sigma–Aldrich) supplemented with 10% FBS (Gibco). Subsequently, 200 μl of resuspended cells were seeded in a 12-well culture plate (Corning Life Sciences) with 800 μl medium. The cells were allowed to adhere for 4 h at 37°C with 5% CO_2_ and observed under a light microscope (DSX510, Olympus, Japan). Then, the non-adherent cells were removed. The purity of the AMs was confirmed by flow cytometry with anti-CD14 antibody.

### Preparation of rat medicated serum

The rat medicated serum was prepared according to the previous protocols [21]. Briefly, 24 SD rats, aged between 6 and 8 weeks and weighing between 180 and 220 g, were divided into four groups, with six rats in each group: NC group, LWBQ low group, LWBQ mid group, and LWBQ high group. The rats in the LWBQ capsule groups were administrated via gastric perfusion of 0.2 g/kg (low dose), 0.4 g/kg (mid dose), and 0.8 g/kg (high dose) LWBQ capsule once a day for 3 days, whereas the NC group was treated with the same volume of 0.9% physiological saline. Blood was aseptically obtained from the abdominal aorta of the rats 1 h after the final administration, and the serum was isolated by centrifugation of the blood at 720×***g*** for 20 min. Following two rounds of filtration using a 0.22-µm cellulose acetate membrane, the serum was bottled, calefied in water at 56°C for 30 min, and then preserved at –80°C for future use.

### Cell grouping and treatment

Cigarette smoke extract (CSE) was prepared as previously reported [22]. In brief, CSE was prepared by bubbling smoke from two cigarettes into 20 ml of serum-free RPMI-1640 and sterile-filtered with a 0.2-μm filter. An optical density of 0.65 (320 nm) was considered to represent 100% CSE and was diluted in serum-free DMEM to 2% CSE. Then AMs were divided into five groups, including NC group, MC group, LWBQ low group, LWBQ mid group, and LWBQ high group. Apart from the NC group, the cells were stimulated with CSE associated with LPS (0.1 µg/ml) for 24 h. After that, the medium was removed and cells were incubated with 10% blank serum or 10% LWBQ-medicated serum for 24 h. At the end of the incubation period, cells were harvested and stored at –80°C for protein and RNA isolation.

### Cytokine analysis

The levels of tumor necrosis factor-α (TNF-α) and interleukin-6 (IL-6) in serum or in culture medium of AMs were measured by ELISA using respective kits (CUSABIO, Wuhan, China) according to the manufacturer’s instructions.

### MTT assay

Cell viability was tested with the MTT assay. Cells were seeded into 96-well plates with 2000 cells/well. Cell viability was assessed using the Vybrant MTT Proliferation Assay Kit (Invitrogen) according to the manufacturer’s instructions. Absorbance was read in a spectrophotometer at a wavelength of 570 nm.

### Assessment of apoptosis by flow cytometry

Cell apoptosis was detected in accordance with the Annexin V/propidium iodide (PI) apoptosis Kit (BioVision, U.S.A.). In brief, 4 × 10^5^ cells were added in each tube. Subsequently, 5 µl Annexin V-fluorescein isothiocyanate and 10 µl PI were added. After mixing, the tube was incubated at 37°C for 15 min in the dark. Analysis was performed using a FACSCalibur flow cytometer.

### Quantitative real-time PCR

Total cellular RNA was isolated from AMs using TRIzol (Invitrogen). cDNA was generated using SuperScript III Reverse Transcriptase (Invitrogen) according to the manufacturer’s instructions. To quantitate the target mRNA, quantitative real-time PCR (qRT-PCR) was performed using the ABI 7500 Real-Time PCR System with SYBR Green I Master (Roche) according to the manufacturer’s instructions. Mean fold-changes were calculated using the 2^−ΔΔ*C*^_t_ method [23]. The relative expression of the genes was normalized to the expression of β-actin. Primers were used as follows:

MMP-9 Forward primer: 5′-GCACGACGTCTTCCAGTACC-3′

MMP-9 Reverse primer: 5′-CAGGATGTCATAGGTCACGTAGC-3′

TIMP1 Forward primer: 5′-TTCTGGCATCCTGTTGTTGCT-3′

TIMP1 Reverse primer: 5′-CCTGATGACGAGGTCGGAATT-3′

β-actin Forward primer: 5′-CCACAGCTGAGAGGGAAATC-3′

β-actin Reverse primer: 5′-TCTCCAGGGAGGAAGAGGAT-3′.

### Western blot

Total protein was extracted from the cells in RIPA lysis buffer as described previously [24]. Equal amounts of cell lysates (40 μg protein) were separated by 10% SDS/PAGE gel, then transferred on to PVDF membranes. Subsequently, the membranes were incubated with primary antibodies including anti-MMP-9 antibody (1:500, Santa Cruz Biotechnology), anti-TIMP1 antibody (1:500, Santa Cruz Biotechnology), and anti-β-actin antibody (1:1000, Sigma–Aldrich) overnight at 4 °C. Then, the membranes were incubated with secondary antibody conjugated to horseradish peroxidase (1:2000; Boster, Wuhan, China) for 1  h at room temperature. The blots were analyzed by chemiluminescence detection (ECL, Amersham).

### Statistical analysis

The data are expressed as the mean ± S.D. All statistical analyses were done using GraphPad Prism 6.0 software (GraphPad Software, California). Comparisons between two groups were evaluated by Student’s *t* test. *P*<0.05 was considered to be statistically significant.

## Results

### Lung function characteristics

**Figure 1:**
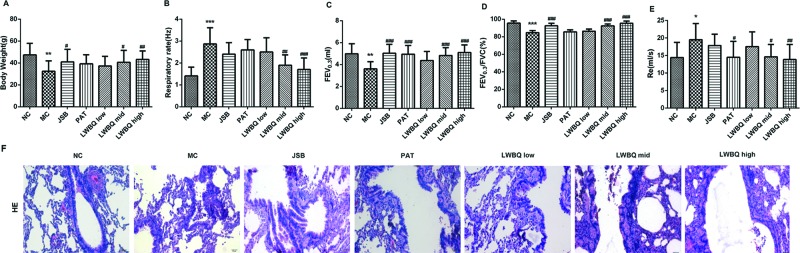
Effects of LWBQ capsule on body weight and lung function parameters in COPD rats (**A**) Body weight (g), (**B**) Respiratory rate (Hz), (**C**) FEV_0.3_, (**D**) ratio of FEV_0.3_ and FVC (FEV_0.3_/FVC), (**E**) Re, (**F**) morphological changes in the lung tissues in COPD rats treated with different drugs (H&E stained, scale bar =20 μm); *P<0.05, **P<0.01, and ***P<0.001 compared with NC group; ^#^*P*<0.05, ^##^*P*<0.01, and ^###^*P*<0.001 compared with MC group.

In the present study, the body weight in the MC group was significantly less than that in the NC group (Figure 1A). Besides, the respiratory rate of the MC rats was notably higher than that of the NC rats (Figure 1B). After different drug treatments, the body weight of rats was heavier and the respiratory rate was lower in comparison with the MC group. Moreover, the body weight and respiratory frequency were better in the LWBQ mid and LWBQ high groups than those in the JSB and PAT groups (Figure 1A,B).

Compared with the NC group, lung function parameters such as FEV_0.3_ and FEV_0.3_/FVC were significantly lower while Re was dramatically higher in the MC rats. On the contrary, FEV_0.3_ and FEV_0.3_/FVC were significantly higher and Re was evidently lower in the LWBQ mid and LWBQ high groups compared with that in the MC group. In addition, the lung function parameters were better in the LWBQ high group than those in the JSB and PAT groups (Figure 1C–E).

### LWBQ capsules improved lung histological condition

The histological analysis of lung tissues showed that the alveolar rupturing, bronchiole stenosis, and the number of the infiltrated inflammatory cells were increased in the MC group compared with NC group. However, these abnormalities were alleviated by different drug treatment (Figure 1F). Especially, airway inflammation was significantly decreased in the LWBQ high group than that in the JSB and PAT groups (Figure 1F).

### Changes in serum cytokines

**Figure 2:**
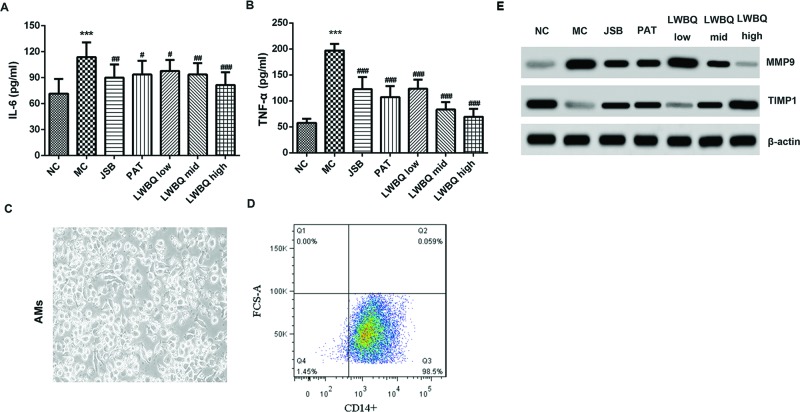
Effects of LWBQ capsule on the release of inflammatory cytokines and the expressions of MMP-9 and TIMP1 in COPD rats (**A**) The levels of IL-6 in the serum were detected by ELISA. (**B**) The levels of TNF-α in the serum were detected by ELISA. (**C**) Morphology of rat AMs after culture for 4 h, magnification ×400. (**D**) Representative images of the purified AMs stained for macrophage marker CD14. (**E**) The protein expressions of MMP-9 and TIMP1 in the AMs from COPD rats were detected by Western blot; ***P<0.001 compared with NC group; ^#^*P*<0.05, ^##^*P*<0.01, and ^###^*P*<0.001 compared with MC group.

Compared with the NC group, the levels of IL-6 and TNF-α were higher in the MC group. After different drug treatments, the levels of IL-6 and TNF-α were significantly reduced compared with the MC group. Besides, LWBQ capsules inhibited the release of IL-6 and TNF-α in a dose-dependent manner (Figure 2A,B). Moreover, the levels of IL-6 and TNF-α were lower in the LWBQ high group compared with the JSB or PAT group.

### Comparison of MMP-9 and TIMP1 protein expression levels in the AMs from COPD rats

AMs are essential for maintaining pulmonary homeostasis [25]. The morphologic analyses of AMs from healthy rats showed that after cultured for 4 h, AMs were round and grew with adherence, indicating that highly purified rat AMs were obtained by adherent culture (Figure 2C). Subsequently, flow cytometry of the macrophage marker CD14 was performed on AMs to assess the purity of the cell population. We found that 98.5% of the cells were CD14^+^ macrophages (Figure 2D).

We evaluated the expression of MMP-9 and TIMP1 in AMs derived from non-COPD and COPD rats. Our results showed that compared with the NC group, MMP-9 protein expression levels were higher while TIMP1 expression levels were lower in the MC group. In contrast, the expression levels of MMP-9 in the LWBQ mid and LWBQ high groups were lower, and TIMP1 was higher than that in the MC group (Figure 2E). In addition, the MMP-9 protein levels in the LWBQ high group were lower, and TIMP1 was higher than that in the JSB or PAT group.

### Effect of LWBQ-medicated serum on cytokine production of AMs

**Figure 3:**
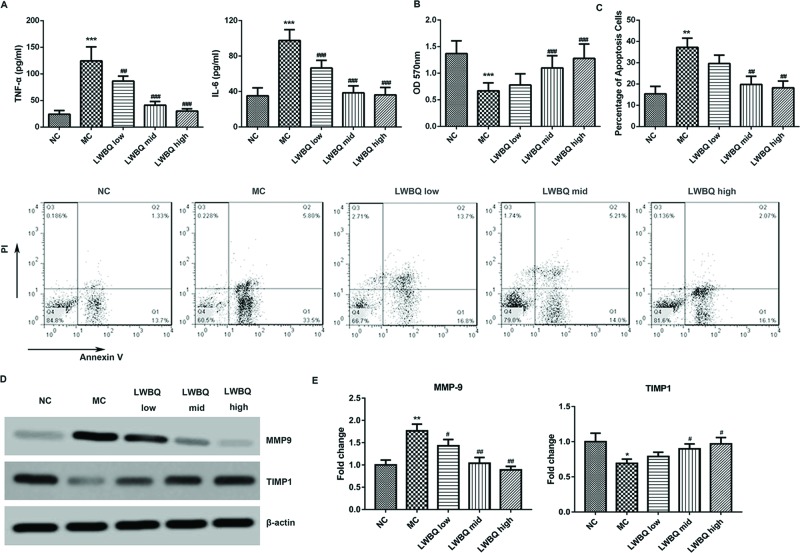
Effect of rat LWBQ-medicated serums on CSE + LPS-induced release of inflammatory cytokines, cell viability, cell apoptosis, and the protein levels of MMP-9 and TIMP1 in AMs (**A**) The levels of TNF-α and IL-6 in the culture medium of AMs. (**B**) The cell viability was measured by MTT method. (**C**) The percentage of apoptotic cells was determined by flow cytometry (FCM). (**D**) The protein expressions of MMP-9 and TIMP1 in the AMs. (**E**) The mRNA expressions of MMP-9 and TIMP1 in the AMs. Abbreviations: MC, model control (CSE + LPS). **P<0.01 and ***P<0.001 compared with NC group, ^#^*P*<0.05, ^##^*P*<0.01, and ^###^*P*<0.001 compared with MC group.

As shown in Figure 3A, compared with NC group, the levels of IL-6 and TNF-α were significantly higher in the MC group. However, the levels of IL-6 and TNF-α were notably decreased by the treatment of LWBQ-medicated serum, when compared with the MC group. In addition, LWBQ-medicated serum dose-dependently down-regulated CSE + LPS-induced up-regulation of IL-6 and TNF-α levels.

### Effect of LWBQ-medicated serum on the viability and apoptosis of AMs

As shown in Figure 3B, after LWBQ-medicated serum treatment, the viability of AMs was significantly higher than that in MC group. Besides, LWBQ-medicated serum dose-dependently increased the viability of AMs. Moreover, the apoptosis of AMs was obviously increased in the MC group compared with that in the NC group (Figure 3C). By contrast, LWBQ-medicated serum displayed more pronounced inhibition effect on cell apoptosis compared with MC group.

### Effect of LWBQ-medicated serum on the MMP-9 and TIMP1 expression levels in AMs

As shown in Figure 3D,E, *MMP-9* mRNA and protein levels were significantly higher while TIMP1 expression levels were clearly lower in the MC group compared with those in NC group. However, a decreased mRNA and protein expression of MMP-9 and an increased expression level of TIMP1 were observed in the AMs after LWBQ-medicated serum treatment compared with those in the MC group.

## Discussion

It has been reported that the JSB capsules can reduce inflammatory response in COPD patients [26]. PAT for the treatment of patients with bronchial asthma can improve the comprehensive immune state of patients [27]. In the present study, we found that the lung function parameters were better and the levels of inflammatory cytokines were lower in the LWBQ high group than those in the JSB and PAT groups, which was in agreement with our previous study [6]. Therefore, LWBQ capsules have better curative effect than other drugs in the treatment of COPD.

The pathophysiology of COPD is multifactorial, which has a link with systemic inflammation with an inflammatory cell profile that includes T lymphocytes macrophages and neutrophils [28,29]. Macrophages, which are derived from monocytes, are thought to be the main mediators of the chronic inflammatory responses seen in patients with COPD [9]. The number of macrophages is increased in the lungs of COPD patients [30]. The pulmonary macrophage system consists of several different populations that are found in alveolar spaces, airways, and resident lung tissue. Besides, AMs constitute over 90% of the pulmonary macrophage population [31]. These cells release a range of proinflammatory mediators [32].

It has been reported that macrophages from patients with COPD release higher levels of proinflammatory cytokines, such as TNF-α and IL-6 compared with non-smoking control subjects [33]. *In vitro*, CSE caused the increased expression of TNF-α and IL-6 in murine AM cell line MH-S [34]. In the present study, we found that the levels of IL-6 and TNF-α were higher in the MC group than those in the normal group. However, LWBQ capsules inhibited the expressions of inflammatory cytokines, which was consistent with previous studies [6].

It is well known that MMP-9 is involved in the pathogenesis of COPD [35]. Previous studies have suggested that MMP-9 and TIMP1 can be secreted by vascular smooth muscle cells [36]. AMs also secrete elastolytic enzymes, such as MMP-9 in response to irritants and infection [28]. Increased MMP-9 activity can augment the degradation of alveolar wall basement membranes, which results in the development of emphysema and airway fibrosis [37]. In addition, MMP-9 overexpression degrades the role of the extracellular matrix and increases airway inflammation [38,39]. TIMP1 acts as an MMP-9 activity inhibitor through binding to its active form and precursors [40]. Cigarette smoke causes MMP/TIMP imbalance [41]. The imbalance between MMP-9 and TIMP1 might result in the degradation of extracellular matrix in pulmonary alveoli, which would cause COPD [42]. The researches also found that the level of MMP-9 was up-regulated, whereas TIMP1 level was down-regulated in lungs of COPD patients compared with control [43]. Our studies demonstrated that MMP-9 expression was higher, and TIMP1 was lower in the MC group compared with the NC group, which was in agreement with previous studies [6].

The present study showed that LWBQ capsules significantly increased TIMP1 expression and reduced MMP-9 expression in AMs. LWBQ capsule is a mixture and the components of LWBQ capsules have been fully explored. According to TCM, Renshen (*Radix Ginseng*) and Huangqi (*Radix Astragali Mongolici*) may nourish vitality. Besides, ginseng saponin can reduce inflammation in LPS-stimulated RAW264.7 cells [44]. Moreover, astragalus polysaccharides and saponins can inhibit MMP-9 expression and enhance TIMP1 expression after cerebral ischemia/reperfusion (I/R) injury in mice [45]. Additional studies have found that astragalus polysaccharide reduces the expression of MMP-9 in rats with COPD and can relieve the lung lesion [46]. The interaction amongst these components of LWBQ capsules plays important roles in alleviating COPD symptoms. Our previous studies have reported that LWBQ capsules suppress inflammation by affecting T-cell polarization and survival in COPD [47]. In the present study, we found that LWBQ capsules can improve COPD symptoms by regulating the expression of MMP-9 and TIMP1.

In conclusion, LWBQ capsules reduce inflammatory response, inhibit cell apoptosis, promote cell viability, and regulate the expression of MMP-9 and TIMP1 in COPD rats. In addition, LWBQ capsules can be a novel therapeutic drug in ameliorating the progression of COPD.

## Abbreviations

AM: alveolar macrophage
BAL: bronchoalveolar lavage
COPD: chronic obstructive pulmonary disease
CSE: cigarette smoke extract
FEV_0.3_: forced expiratory flow in 0.3 s
FVC: forced vital capacity
H&E: hematoxylin and eosin
IL-6: interleukin-6
JSB: Jinshuibao
LPS: lipopolysachharide
LWBQ: Liuweibuqi
LWBQ low: low dose of LWBQ
LWBQ mid: mid dose of LWBQ
LWBQ high: high dose of LWBQ
MC: model control
MMP: matrix metalloproteinase
NC: normal control
PAT: spleen aminopeptidase
PI: propidium iodide
Re: expiratory resistance
SD: Sprague–Dawley
TCM: traditional Chinese medicine
TNF-α: tumor necrosis factor-α
TIMP: tissue inhibitor of metalloproteinases
